# Uncertainty in near-term temperature evolution must not obscure assessments of climate mitigation benefits

**DOI:** 10.1038/s41467-022-31425-x

**Published:** 2022-07-14

**Authors:** Alexandrine Lanson, Peter Pfleiderer, Flavio Lehner, Carl-Friedrich Schleussner

**Affiliations:** 1 Climate Analytics, Berlin, Germany; 2 Integrative Research Institute on Transformations of Human-Environment Systems (IRI THESys) and Geography Department, Humboldt University, Berlin, Germany; 3 Department of Earth and Atmospheric Sciences, Cornell University, Ithaca, NY USA; 4 Climate and Global Dynamics Laboratory, National Center for Atmospheric Research, Boulder, USA; 5 Institute for Atmospheric and Climate Science, ETH Zürich, Zürich, Switzerland

**Keywords:** Attribution, Projection and prediction

**arising from** B. H. Samset et al. *Nature Communications* 10.1038/s41467-020-17001-1 (2020)

In a recent study, Samset et al.^1^ reported that due to the imprint of natural variability, the effects of emission mitigation will only be perceived through global temperature with a multi-decadal delay. Their analysis, also including a decomposition into the effects of mitigating individual climate forcers, is highly relevant and timely, but does not fully substantiate all conclusions made by the authors. Here, we provide additional context around the claims by Samset et al.^1^ of multi-decadal delays of mitigation benefits and express concerns with their conceptual approach towards assessing a discernible warming response under different greenhouse gas concentration pathways. A broader debate on how to best assess and communicate emerging effects of climate mitigation in the light of natural variability seems warranted.

Increased atmospheric greenhouse gas concentrations lead to increased radiative forcing and thus warming^2^. Important differences exist between long- and short-lived climate forcers that need to be taken into account when assessing warming under different emissions trajectories. For CO2 as the dominant greenhouse gas, reduced emissions directly translate into reduced warming^2^. The Representative Concentration Pathways (RCPs) deployed by Samset et al.^1^, are distinct greenhouse gas concentration pathways, and consequently, differences in their mean warming response as modelled by the reduced-complexity climate model MAGICC are evident in Fig. 1 of Samset et al.^1^ within years and even before 2020.

**Fig. 1:**
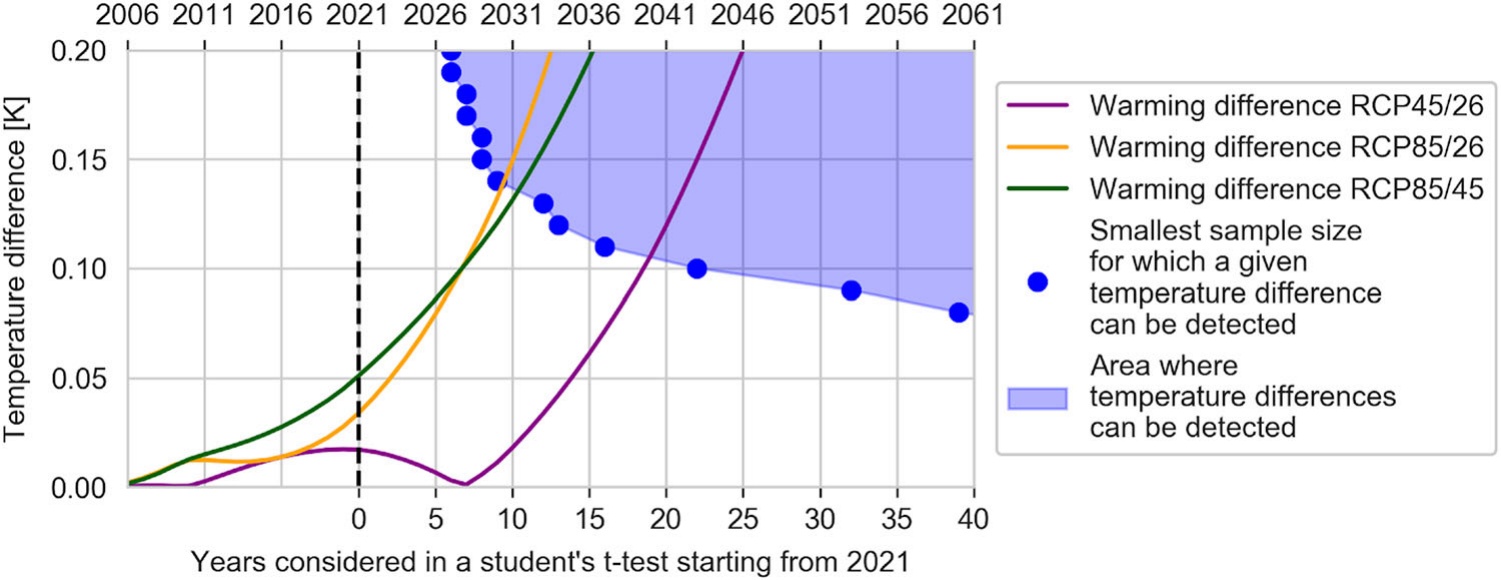
Detectability of temperature differences using the Samset method. For different constant temperature differences and different sample sizes, a Student’s t-test is applied to pairs of annual global mean temperature variability time series from CESM1 LENS^9^. Blue dots indicate the minimal sample size for which a given temperature difference is detected by 66% of the pairs at a 0.05 significance level, the significance threshold chosen by Samset et al.^1^ The area right of the blue dots is the area for which temperature differences can be detected. Solid lines show emerging warming differences in the forced global mean temperature (GMT) response between different Representative Concentration Pathways (RCPs, from MAGICC6).

Why is it then that Samset et al.^1^ conclude that it may take decades for the effects of mitigation to emerge? The explanation lies in the imprint of natural variability on the near-term temperature trajectory and their assessment of its effects. Natural variability, including multi-decadal modes linked to ocean dynamics, dominates the uncertainty of global mean temperature evolution on decadal timescales^3^. Thus, the effects of mitigation may not be immediately perceived when assessing a single GMT trajectory on short time scales. This is the concern expressed by Samset et al.^1^ with respect to the public perception of the immediate benefits of climate action.

The results and interpretations of Samset et al.^1^ rely on their conception of emergence following an approach by Tebaldi and Friedlingstein^4^. They define the year of emergence of a significant signal as “the first year when at least 66% of the baseline-scenario pairs are statistically significantly different”^1^ (baseline-scenarios pairs being RCP4.5-mitigation scenario pairs), using a Student’s *t*-test (*p* < 0.05).

The methodological choice needs to be critically reviewed in light of the question it tries to address. Rather than testing for the effects of mitigation on a given warming trajectory, this test assesses when any possible GMT trajectory under a mitigation scenario would be discernible from any possible GMT evolution under a reference scenario (or 66% of those randomly combined samples). This is very different from assessing the actual effects of mitigation on an individual trajectory, or the ensemble response. Naturally, robust differences in such a test will only emerge after the mitigation signal dominates over natural variability. Samset et al.^1^ find that it requires about 0.2 °C of anthropogenic warming difference for this test to yield robust results of emergence. Discernible differences in climate impacts such as extreme temperature or long-term sea level rise can already be detected for similar GMT differences^5^.

The authors argue that their approach is the appropriate way to assess the question of emergence as “[the] emergence of a climate mitigation signal beyond natural variability can never be proven, as we would be comparing to an unknown, counterfactual world*.”*^1^ This assertion is at least debatable. A range of well-established approaches exist to assess the anthropogenic warming contribution in the presence of natural variability^3,6^. Therefore, at any given point in time, we are able to assess the effects of mitigation on the anthropogenic warming trend. Uncertainty around this assessment will be much smaller than the irreducible uncertainty portrayed in Samset et al.^1^. It is also worth recalling that the Paris Agreement refers only to anthropogenic climate change and excludes natural variability^7^. Therefore, assessments of “the progress made towards the ambitions of the Paris Agreement”^1^ would not need to rely on approaches such as the one proposed by Samset et al.^1^.

Furthermore, short-term warming trends under different emissions scenarios affected by natural variability can be decomposed to reconcile observed and modelled warming trends. The scientific community has done so extensively when assessing the so-called warming hiatus period in the early twenty-first century, during which observed GMT increase slowed, and identified the contribution of natural variability in explaining this short-term slow-down^8^. There is no reason to believe that this will not be possible going forward. Indeed, different strands of detection and attribution research, such as on extreme weather events, are commonly dealing with even stronger presence of natural variability and are perfectly able to quantify partial contributions of anthropogenic climate change^9^. For example, 2021 featured an outsized number of devastating extreme events despite its GMT being colder than 5 of the last 6 years^10^. Careful explanation of such apparent contradictions between a common warming target (GMT) and impacts (extreme events in populated areas) is critical to inform the public on what they can expect from mitigation^11^.

The approach applied by Samset et al.^1^ has limitations with respect to the detectability of an emergent climate signal as it does not allow for a clear distinction between two factors influencing the Student’s t-test’s significance: (1) the magnitude of the forced warming response and (2) the sample size (time series length). While (1) is the signal that Samset et al.^1^ are interested in, (2) grows over time and thereby unintentionally influences the significance testing. In their analysis, Samset et al.^1^ assess GMT trajectories from 2021 onward. Thus, the Student’s t-test is performed with very small sample sizes in the near future. We have illustrated the effects of warming difference and sample size in Fig. 1 for constant warming differences. We find that a minimum of about 10 years (which would be well after 2030 for an annual time series) is required to robustly detect a constant 0.15 °C temperature difference. For an emerging warming difference between scenarios over time (see Fig. 1), robust detection will only be possible considerably later. The core findings of Samset et al.^1^ of a delayed emergence of robust differences between the RCPs therefore depend at least in part on statistical effects resulting from their methodological choices rather than climate system uncertainty. Note that this short-coming is not specifically linked to the Student’s t-test, but applies more generally to any approach based on mere statistical comparison of individual time series.

This serves as an illustration of how much methodological choices affect the outcome of such studies and how they require very careful communication and explanation. Policy makers or the general public may not understand the implications of different approaches. This is even more relevant as messaging around these issues relates to one of the core challenges of addressing climate change as a collective action problem: that the biggest benefits of rapid mitigation action in terms of avoided climate impacts lie in the future. However, robust differences in warming rates as a result of stringent mitigation are observable already in the near-term^12^, and such a slow-down in warming might have concrete benefits for building climate resilience in the next decades, particularly in developing countries^13^. Holistic communication on the benefits of climate action should thereby also include an assessment of the co-benefits of mitigation, such as reduced air pollution, and related health benefits^14^. Lastly, lessons on how to communicate benefits of climate action in the absence of real-world counterfactuals may draw on lessons learned from other disciplines. The experience of epidemiological modelling of the COVID-19 Pandemic and the assessment of the effect of specific responses may provide for some useful comparison here^15^. Specifically, reflections on how to communicate ‘self-defeating’ forecasts might help to advance the debate:^16^ This can inform communication strategies around benefits that arise from societies responding upon a forecast with preventive measures, thereby falsifying the worst-case outcome. Irreducible uncertainty in near-term climate projections must not obscure the messaging around our understanding of the response of the climate system to reducing greenhouse gas emissions.

## Data Availability

All data generated or analysed during this study are included in the published article in ref. ^9^. The code underlying Fig. 1 is available from the authors upon reasonable request.
